# How do individuals rate their health compared to others? findings based on a nationally representative sample in Germany

**DOI:** 10.1186/s12889-023-17600-9

**Published:** 2024-01-16

**Authors:** André Hajek, Hans-Helmut König

**Affiliations:** Department of Health Economics and Health Services Research, University Medical Center Hamburg-Eppendorf, Hamburg Center for Health Economics, Hamburg, Germany

**Keywords:** Health comparisons, Sports, Alcohol, Meat, Lifestyle, Income

## Abstract

**Background:**

The aim of this study is to explore the determinants of health comparisons (i.e., how individuals rate their health compared to other individuals in their age bracket) in the general adult population (total sample and in different age groups).

**Methods:**

Data were used from the general adult population in Germany (wave 46, n = 3,876 individuals; November 2021 to January 2022), based on the GESIS panel, which is a probability-based mixed-mode panel. Health comparisons were used as outcome measure. Socioeconomic, lifestyle-related and health-related determinants were included in regression analysis. Robustness checks were conducted.

**Results:**

Regressions showed that more favorable health comparisons were associated with being male (among individuals up to 39 years), higher age (among the total sample), higher education (among the total sample and individuals up to 39 years), higher income (among the total sample and individuals aged 40 to 64 years), not “being married, and living together with a spouse” (among the total sample), never eating meat (among the total sample, individuals up to 39 years and particularly individuals aged 40 to 64 years), drinking alcohol (among the total sample, individuals aged 40 to 64 years and individuals aged 65 years and over), a higher frequency of sports activities (all groups) and a higher satisfaction with health (also in all groups).

**Conclusion:**

In addition to the evident link between health satisfaction and health comparisons, regression analysis revealed that certain socioeconomic factors, such as a higher income level, along with positive lifestyle-related factors - especially among middle-aged individuals - were significantly associated with more positive health comparisons. This knowledge is required in order to support individuals at risk for negative health comparisons. This is important because negative health comparisons can contribute to poor well-being and poor health outcomes.

**Supplementary Information:**

The online version contains supplementary material available at 10.1186/s12889-023-17600-9.

## Background

The primary concept underlying this study is based on the Easterlin Paradox; a famous concept in the field of happiness economics. There is an observed association between income and subjective well-being (SWB) in cross-sectional studies. However there is not a clear association between income and SWB longitudinally [1, 2]. In other words: While individuals earning more report a higher SWB than their lower-income counterparts at a given point in time, higher incomes do not produce greater SWB over time. This phenomenon is known as the “Easterlin Paradox”.

One possible explanation for this paradox is the importance of relative income. Namely, when the income of others rises, one’s own increased income initially contributes to increases in SWB since one may not be aware of the increased income of others. However, over time, one may be aware of the increased income of others, and the rise in SWB stemming from the increase in one’s own income diminishes. Thus, the Easterlin paradox stems from the contradiction between cross-sectional studies and long-term trends: In the short term, there seems to be an association between rising income and SWB, which leads individuals to strive for higher incomes. In the long run, however, SWB usually does not increase as the efforts of others to improve their income can contribute to higher average incomes, ultimately leaving everyone in a similar position when referring to their relative income. Relative income levels, in relation to reference groups such as co-workers, or individuals with a similar education background, are considered to contribute to overall wellbeing, as shown by Ferrer-i-Carbonell [3].

Previous studies have suggested that such comparisons are not limited to income alone, but have a more general nature and can thus refer to age or even health comparisons [4]. In other words: similar to income where we can distinguish between actual income and income comparisons (relative income), we can distinguish between actual health (e.g., chronic conditions or self-rated health) and comparisons in relation to health.

In fact, a few recent studies have shown that health comparisons are important for several important outcomes [5–8]. Specifically, one study has shown that negative health comparisons in particular (when one’s own health is considered worse compared to other individuals in one’s own age bracket) are relevant for unfavorable SWB among community-dwelling individuals aged 40 years and over in Germany [5] based on nationally representative longitudinal data from the German Ageing Survey. Another study showed that the resilience factors of optimism, self-esteem, and self-efficacy may help mitigate the adverse effects of negative health comparisons for SWB [9]. A further study showed that negative health comparisons can contribute to poor functional health among men, and more depressive symptoms among women, based on German data [6]. Additionally, recent research demonstrated that negative health comparisons are associated with higher loneliness levels [10]. Moreover, both negative and positive health comparisons hold particular significance in relation to the perception of social isolation [7, 10] (based on studies conducted in Germany). In view of these findings, it is important to clarify the determinants of negative or positive health comparisons.

The association between, for example, socio-economic status (e.g., asset, income or education) and health has been thoroughly studied; emphasizing the relevance of socio-economic factors in influencing one’s *health* in terms of numerous chronic conditions, self-rated health or mortality [11, 12]. However, there have not been any studies conducted to date that investigate the determinants of *health comparisons*. Thus, we aimed to identify the determinants of health comparisons based on a representative sample of the general adult population in Germany. Gaining insights into the factors associated with unfavorable health comparisons can aid in identifying individuals who may be at risk for such negative comparisons. This is important because, as noted above, unfavorable health comparisons can contribute to low SWB, isolation and poor health outcomes.

## Methods

### Sample

Data were taken from the GESIS panel. This is a probability-based mixed-mode panel of the general adult population in Germany. It includes multiple topics of general interest. In the GESIS panel, the reference population is the German-speaking population aged 18 to 70 years and permanently living in Germany.

First, a random sample was drawn from municipal population registers. Based on this random sample, 4,938 panelists, were recruited in the year 2013. The response rate was 86%. Two self-administered survey modes (i.e., online and paper-and-pencil) were applied for the initial profile survey and all following waves. The following waves were conducted bi-monthly (2013–2020) or tri-monthly (2021 onwards). For the GESIS panel, the completion rates per wave were approximately 90% of invited participants (online mode) or 85% (off-line mode). More details are given elsewhere [13].

The GESIS panel is open for data collection for the academic research community. Thus, it includes key questions such as sociodemographic data, and tools that have been developed by the scientific community studying political science, psychology, economics or sociology. These tools are evaluated based on a review process. This review process is internal or external, depending on the length of the tools. Further details are given elsewhere [14]. The authors of this study proposed the outcome of this study to the GESIS panel. After a successful internal review process on the part of GESIS, the outcome was included in wave 46 of the GESIS panel.

Due to reasons of data availability, data were used from wave 46 of the GESIS panel, which took place from November 2021 to January 2022. In total, 3,876 individuals gave answers relating to the dependent variable, and 48 individuals did not want to answer this question. In the first analytical sample, n equaled 3,222, when listwise deletion was used to address missing data. In the second analytical sample, n equaled 3,876 individuals, when full-information maximum likelihood (FIML) was used to address missing data.

### Outcome

Health comparisons were quantified using the following question: “How would you rate your health compared with other people your age?” (much better; somewhat better; the same; somewhat worse; much worse). This is a common way to quantify health comparisons (e.g., [8]). To ease the interpretation of the findings, we recoded the variable: from 1 = much worse to 5 = much better.

### Independent variables

In the regression analysis, a range of socioeconomic, lifestyle, and health-related factors were chosen as independent variables. Due to the limited number of studies in this area, the independent variables were mainly selected on the basis of theoretical considerations. For example, it may be the case that women and men differ in their health comparisons because they may differ in their evaluation of their own health [15]. For the same reason, different age groups could also differ in their health comparisons [15]. We also assumed that people who are active in sports differ from people who are less active in terms of health comparisons, as more active people may be aware of the positive impact of sports activities [16]. The same applies to other lifestyle factors such as meat consumption and alcohol consumption. It also seems plausible that satisfaction with one’s own health can contribute to health comparisons, as health factors can certainly have an immediate impact on the assessment of one’s own health [15].

With regard to socioeconomic factors, we used sex (men; women), age (in years; extreme values (i.e., “born 1995 or later” to 1995; “born 1943 or earlier” to 1943) were adapted to remain anonymity), marital status (dichotomized into: married, living together with spouse and others (i.e., married, living separated from spouse, widowed, single, divorced), education and income. Education was dichotomized using a median-split. Lower school education refers to: (i) still a student and attending a general school, (ii) left school without obtaining a degree, (iii) completion of education within a maximum of 7 years (abroad), (iv) Polytechnic Secondary School of the German Democratic Republic (GDR) with completion after the 8th or 9th grade, (v) Polytechnic Secondary School of the German Democratic Republic (GDR) with completion after the 10th grade, (vi) completion of basic school education, primary school diploma, (vii) completion of secondary school education, intermediate level. Higher school education refers to: (i) Completion of technical college entrance qualification, and (ii) General or subject-specific university entrance qualification (secondary school or specialized secondary school, including specialized secondary school with vocational training). The average household net income per month in Euro (9 categories from “below 900 Euro” to “6000 Euro and more”) was also dichotomized using a median-split: below median: below 3200 Euro; above median: 3200 Euro or more.

With regard to lifestyle-factors, we included: meat consumption in the past four weeks, alcohol consumption (beer, wine, or spirits) in the past four weeks, and frequency of sports activities such as jogging or fitness in the past four weeks. In each case, the answer categories were: never, about 1 time a month, 2–3 times a month, 1–2 times per week, 3–4 times per week, every day or almost every day. Furthermore, with regard to health, we included satisfaction with overall health (single item ranging from 1 = very unsatisfied to 7 = very satisfied).

### Statistical analysis

Sample characteristics are displayed by ratings of health comparisons (much worse, somewhat worse, the same, somewhat better, much better). Thereafter, multiple linear regressions (total sample and stratified by age group) were performed to explore the determinants of health comparisons. In regression analysis, health comparisons was used as dependent variable. As independent variables, we used sex, age, education, income, marital status, meat consumption, alcohol consumption, sports activities, and satisfaction with health. Robust standard errors were calculated. In a first sensitivity analysis, ordered probit regressions were used to consider the potential ordinal nature of the dependent variable. Moreover, a FIML approach was used to deal with missing values in further sensitivity analysis [17]. The statistical significance was determined with *p* < 0.05. Stata 16.1 was used for statistical analysis (StataCorp, College Station, TX, USA).

## Results

### Sample characteristics

Sample characteristics are shown in Table 1. Overall, among the respondents, 10.2% reported their health to be much better than others in their age group, whereas 28.3% rated it as somewhat better. Furthermore, 38.8% indicated that their health was the same. In contrast, 17.8% felt their health was somewhat worse, and 4.9% considered it to be much worse. Average age equaled 57.2 years (SD: 14.2 years) and 49.3% were female. Health comparisons significantly differed according to sex, age, school education, household net income, meat consumption, alcohol consumption, frequency of sports activities and satisfaction with health. However, the outcome measure was not significantly associated with marital status. Additional details are provided in Table 1.

**Table 1:** Sample characteristics (also stratified by health comparisons)

Variables	Much worse	Somewhat worse	The same	Somewhat better	Much better	Total	*P*-value
n (%)	189 (4.9)	690 (17.8)	1,505 (38.8)	1,095 (28.3)	397 (10.2)	3,876 (100.0)	
Sex, n (%)							0.043
Men	103 (55.1)	334 (48.7)	740 (49.4)	589 (54.1)	190 (47.9)	1,956 (50.7)	
Women	84 (44.9)	352 (51.3)	758 (50.6)	500 (45.9)	207 (52.1)	1,901 (49.3)	
Age, mean (sd)	60.1 (12.2)	55.7 (14.1)	55.2 (14.3)	58.4 (14.2)	62.7 (12.9)	57.2 (14.2)	< 0.001
School education, n (%)							< 0.001
Lower than technical college entrance qualification	134 (72.8)	330 (48.5)	740 (49.6)	469 (43.0)	194 (49.6)	1867 (48.7)	
Technical college entrance qualification or higher	50 (27.2)	350 (51.5)	752 (50.4)	621 (57.0)	197 (50.4)	1970 (51.3)	
Household net income, n (%)							< 0.001
Below median	106 (65.4)	308 (50.1)	572 (42.8)	375 (37.8)	153 (42.5)	1514 (43.7)	
Above median	56 (34.6)	307 (49.9)	765 (57.2)	618 (62.2)	207 (57.5)	1953 (56.3)	
Marital status, n (%)							0.107
Single/Widowed/Divorced/Married, not living together with spouse	73 (39.0)	268 (39.2)	507 (33.9)	373 (34.2)	142 (35.8)	1363 (35.3)	
Married, living together with spouse	114 (61.0)	416 (60.8)	990 (66.1)	718 (65.8)	255 (64.2)	2493 (64.7)	
Meat consumption, n (%)							< 0.001
Never	6 (3.2)	29 (4.2)	39 (2.6)	51 (4.7)	31 (7.9)	156 (4.1)	
About 1 time a month	12 (6.4)	29 (4.2)	28 (1.9)	42 (3.9)	17 (4.3)	128 (3.3)	
2–3 times a month	27 (14.4)	86 (12.6)	149 (10.0)	132 (12.2)	47 (12.0)	441 (11.5)	
1–2 times per week	68 (36.4)	213 (31.1)	572 (38.4)	400 (36.8)	162 (41.3)	1415 (36.8)	
3–4 times per week	49 (26.2)	199 (29.1)	459 (30.8)	334 (30.8)	96 (24.5)	1137 (29.6)	
Every day or almost every day	25 (13.4)	128 (18.7)	244 (16.4)	127 (11.7)	39 (9.9)	563 (14.7)	
Alcohol consumption, n (%)							< 0.001
Never	71 (37.8)	165 (24.2)	233 (15.6)	142 (13.1)	62 (15.8)	673 (17.5)	
About 1 time a month	27 (14.4)	102 (15.0)	193 (12.9)	132 (12.2)	51 (13.0)	505 (13.2)	
2–3 times a month	23 (12.2)	112 (16.4)	261 (17.5)	184 (17.0)	62 (15.8)	642 (16.7)	
1–2 times per week	37 (19.7)	139 (20.4)	401 (26.9)	306 (28.2)	102 (26.0)	985 (25.7)	
3–4 times per week	15 (8.0)	94 (13.8)	288 (19.3)	204 (18.8)	67 (17.0)	668 (17.4)	
Every day or almost every day	15 (8.0)	70 (10.3)	116 (7.8)	117 (10.8)	49 (12.5)	367 (9.6)	
Frequency of sports activities, n (%)							< 0.001
Never	80 (43.2)	221 (33.1)	405 (27.4)	165 (15.2)	68 (17.4)	939 (24.7)	
About 1 time a month	27 (14.6)	95 (14.2)	183 (12.4)	80 (7.4)	21 (5.4)	406 (10.7)	
2–3 times a month	10 (5.4)	83 (12.4)	203 (13.7)	135 (12.5)	29 (7.4)	460 (12.1)	
1–2 times per week	41 (22.2)	163 (24.4)	421 (28.5)	380 (35.1)	114 (29.2)	1119 (29.4)	
3–4 times per week	11 (5.9)	69 (10.3)	167 (11.3)	204 (18.9)	86 (22.1)	537 (14.1)	
Every day or almost every day	16 (8.6)	36 (5.4)	99 (6.7)	118 (10.9)	72 (18.5)	341 (9.0)	
Satisfaction with health (from 1 = very unsatisfied to 7 = very satisfied), mean (sd)	2.9 (1.7)	4.2 (1.3)	5.2 (1.2)	5.5 (1.1)	5.9 (1.1)	5.1 (1.4)	< 0.001

Notes: *p*-values are based on Chi²-tests or oneway ANOVAs, as appropriate

### Regression analysis

In Table 2, results of multiple linear regressions are given (second column: among the total sample; third, fourth and fifth column: among individuals aged up to 39 years, 40 to 64 years and 65 years and over, respectively).

**Table 2:** Determinants of health comparisons. Results of multiple linear regressions (with listwise deletion to address missings)

Independent variables	Health comparisons– Total sample	Health comparisons– up to 39 years	Health comparisons– 40 to 64 years	Health comparisons– 65 years and over
Sex: Female (Ref.: Male)	-0.00	-0.20*	-0.03	0.11+
	(0.03)	(0.08)	(0.04)	(0.06)
Age in years	0.01***	0.00	0.01+	0.01+
	(0.00)	(0.01)	(0.00)	(0.01)
Education: Technical college entrance qualification or higher (Ref.: Lower than technical college entrance qualification)	0.08*	0.20*	0.05	0.08
	(0.03)	(0.09)	(0.04)	(0.06)
Household net income: above median (Ref.: below median)	0.13***	0.10	0.16**	0.11+
	(0.03)	(0.08)	(0.05)	(0.06)
Marital status: Married, living together with spouse (Ref.: Single/Widowed/Divorced/Married, not living together with spouse)	-0.10**	-0.05	-0.09+	-0.10
	(0.03)	(0.08)	(0.05)	(0.07)
Meat consumption: - About 1 time a month (Ref.: Never)	-0.22+	-0.45*	-0.37*	0.22
	(0.12)	(0.22)	(0.18)	(0.23)
− 2–3 times a month	-0.32***	-0.36*	-0.36*	-0.28
	(0.09)	(0.16)	(0.14)	(0.20)
− 1–2 times per week	-0.26**	-0.20	-0.36**	-0.22
	(0.08)	(0.14)	(0.13)	(0.19)
− 3–4 times per week	-0.27**	-0.13	-0.35**	-0.24
	(0.08)	(0.14)	(0.13)	(0.19)
- Every day or almost every day	-0.36***	-0.20	-0.44**	-0.38+
	(0.09)	(0.15)	(0.14)	(0.21)
Alcohol consumption: - About 1 time a month (Ref.: Never)	0.17**	0.03	0.24**	0.12
	(0.06)	(0.12)	(0.08)	(0.11)
− 2–3 times a month	0.15**	-0.02	0.18*	0.21*
	(0.05)	(0.11)	(0.07)	(0.11)
− 1–2 times per week	0.22***	0.10	0.24***	0.24**
	(0.05)	(0.10)	(0.07)	(0.09)
− 3–4 times per week	0.18***	-0.04	0.20**	0.25*
	(0.05)	(0.13)	(0.07)	(0.10)
- Every day or almost every day	0.18**	0.17	0.18+	0.28*
	(0.07)	(0.22)	(0.09)	(0.11)
Frequency of sports activities: - About 1 time a month (Ref.: Never)	0.02	-0.32**	0.14*	-0.00
	(0.05)	(0.11)	(0.07)	(0.11)
− 2–3 times a month	0.14**	-0.04	0.31***	-0.05
	(0.05)	(0.11)	(0.07)	(0.10)
− 1–2 times per week	0.26***	0.08	0.34***	0.17*
	(0.04)	(0.10)	(0.06)	(0.08)
− 3–4 times per week	0.39***	0.38**	0.51***	0.21*
	(0.05)	(0.13)	(0.07)	(0.09)
- Every day or almost every day	0.49***	0.33*	0.65***	0.33**
	(0.06)	(0.16)	(0.10)	(0.10)
Satisfaction with health (from 1 = very unsatisfied to 7 = very satisfied)	0.32***	0.30***	0.31***	0.34***
	(0.01)	(0.03)	(0.02)	(0.02)
Constant	0.82***	1.45***	1.13***	0.76
	(0.13)	(0.40)	(0.24)	(0.53)
Observations	3,222	459	1,651	1,112
R²	0.29	0.34	0.31	0.25

Beta-coefficients (unstandardized) are displayed; robust standard errors in parentheses; *** *p* < 0.001, ** *p* < 0.01, * *p* < 0.05, + *p* < 0.10

More favorable health comparisons were associated with being male (among individuals up to 39 years), higher age (among the total sample), higher education (among the total sample and individuals up to 39 years), higher income (among the total sample and individuals aged 40 to 64 years), not “being married, and living together with a spouse” (among the total sample), never eating meat (among the total sample, individuals up to 39 years and particularly individuals aged 40 to 64 years), drinking alcohol (among the total sample, individuals aged 40 to 64 years and individuals aged 65 years and over), a higher frequency of sports activities (all groups) and a higher satisfaction with health (also in all groups).

In sensitivity analysis, ordered probit regressions were used instead of linear regressions; please see Supplementary Table 1. In terms of significance, results remained virtually the same. Moreover, in further sensitivity analysis, a FIML approach was used to tackle missing data instead of listwise deletion. Again, the results remained nearly unchanged compared to our main model. The results are shown in Supplementary Table 2.

## Discussion

### Key findings

The objective of this study was to examine the determinants of health comparisons in the German population. Beyond the obvious association between satisfaction with health and health comparisons, some socioeconomic factors such as higher education and higher income, as well as favorable lifestyle-related factors, particularly among individuals aged 40 to 64 years, were significantly associated with more favorable health comparisons in regression analysis. This present study contributes novel insights into our understanding of health comparisons and expands our current knowledge in this area.

### Prior research and possible explanations

It is challenging to compare our present findings with previous studies, as there are no studies that have examined the determinants of health comparisons. Therefore, in this section, we will mainly focus on the determinants identified among the total sample, but will also briefly mention notable findings among the subgroups.

We identified an association between higher age and favorable health comparisons. Possible explanations could be that older people are more likely to compare themselves with their peers (of the same age group) who are in poorer health. Older people could also have lower expectations regarding their own health [18]. These factors could lead to more positive health comparisons in later life.

Moreover, our study showed that favorable health comparisons were present among individuals with higher education and individuals with a higher income level. For example, individuals with higher education and higher income may compare themselves with other individuals in their age bracket, such as former classmates from primary school with lower school education, or with colleagues with lower income. Thus, they may feel that they are better off– e.g., in terms of income [3] or even health. Moreover, such individuals with high education and high income may have a greater health awareness and greater health-related knowledge. The aforementioned association between higher education/income and higher health awareness/knowledge has been shown by various previous studies (e.g., [19–22]). This may be a key reason why well-educated and more affluent individuals report more favorable health comparisons.

Individuals not belonging to the group “being married and living together with a spouse” showed more favorable health comparisons. One way to explain such findings is that such individuals may have significantly more free time available, due to fewer or no family commitments, compared to other married individuals, who take care of their children and their parents [23]. This free time could, in turn, be invested in health-promoting measures, which could promote positive health comparisons. An association between being unmarried and a higher likelihood of sports activities has been identified in prior research [24–26]. This association has been explained by the lesser family obligations and greater leisure time [24–26].

Never eating meat was associated with favorable health comparisons in our study, particularly among the middle-aged. Never eating meat in the past four weeks may reflect healthier eating behaviors in general. For example, individuals not eating meat may think that they are better off in terms of health compared to individuals who eat meat daily, as eating certain types of meat - such as red meat - is associated with, among other things, some cardiovascular risk factors [27] and various cancer types [28]. Thus, it may be a question of attitude, i.e., individuals may feel better because they think that avoiding meat is good for their health.

In the total sample, and in all age groups, individuals who undertook frequent sports activities had favorable health comparisons in our study. On the one hand, this can be explained by the fact that active people are also aware of the benefits of physical activity for the body [16]. They may also notice that they are fitter than other people in their age group, e.g. on the way to lunch at work when climbing stairs, or comparing themselves with friends of the same age. Equally, they may feel as if they are a role model for peers [29]. Moreover, some individuals may be more active *because* they have better overall health, which in turn could be associated with a more favorable health comparison. Overall, sport seems to play a central role for health comparisons at any age.

At first glance, it is surprising that never drinkers reported unfavorable health comparisons. One potential explanation: Those who drink wine, for example, may have supposed health benefits in mind [30]. However, it should be acknowledged that the category of never drinkers can contain ascetics, as well as potentially former alcoholics, i.e., ex-drinkers may reflect a notable proportion of current never drinkers. Some ex-drinkers may abstain from alcohol for health reasons. In this respect, abstaining from alcohol could also reflect a poor state of health. With comparable instruments for measuring alcohol consumption [31], this can lead to potentially invalid conclusions. In this respect, these results should be interpreted with great caution and further research is urgently needed.

### Strengths and limitations

Some strengths and limitations should be taking into account when interpreting our results. We used data from the general adult population in Germany. Moreover, sensitivity analyses were performed to ensure the robustness of our results. Of note, our study explicitly refers to one’s age group when making health comparisons; which is most likely the most important reference group when making health comparisons. However, other factors may also be of importance when making health comparisons, such as neighborhoods or social networks. Furthermore, it is worth noting that this is a cross-sectional study which makes it difficult to clarify the directionality of the associations. Moreover, former research demonstrated that the monolingual character of the GESIS panel could contribute to selective drop-out in non-native individuals [13]. Additionally, another limitation is that chronic illnesses were not quantified in the data used. Moreover, a potential bias due to common method variance (e.g., with satisfaction with health) should be acknowledged [32]. Furthermore, some panel attrition has been detected in the GESIS panel (about 10–12% per year) [33],which is comparable to other large panels such as the German Socio-Economic Panel (SOEP) [34].

## Conclusion

In addition to the evident link between health satisfaction and health comparisons, regression analysis revealed that certain socioeconomic factors, such as a higher income level, along with positive lifestyle-related factors, especially among middle-aged individuals, were significantly associated with more positive health comparisons. Such knowledge is important to address individuals at risk for negative health comparisons. Upcoming research in this neglected research area is recommended. For example, having children may play a role in explaining the association between marital status and health comparison, and thus could be further explored.

### Electronic supplementary material

Below is the link to the electronic supplementary material.

Supplementary Material 1

## Data Availability

Data are available for scientific use on the website: https://www.gesis.org/en/gesis-panel/data.

## Abbreviations

FIML: full-information maximum likelihood
GDR: German Democratic Republic
SWB: Subjective well-being
