# Supplementary material for: How do individuals rate their health compared to others? findings based on a nationally representative sample in Germany
Source: BMC Public Health. 2024 Jan 16;24:197. doi: 10.1186/s12889-023-17600-9 (PMC10792948; doi:10.1186/s12889-023-17600-9)
Supplement: Supplementary file 1 — Supplementary Material 1 [file 12889_2023_17600_MOESM1_ESM.docx]

Supplementary Table 1. Correlates of health comparisons. Results of ordered probit regressions

| Independent variables | Health comparisons – Total sample | Health comparisons – up to 39 years | Health comparisons – 40 to 64 years | Health comparisons – 65 years and over |
| --- | --- | --- | --- | --- |
|  |  |  |  |  |
| Sex: Female (Ref.: Male) | -0.00 | -0.30** | -0.04 | 0.13+ |
|  | (0.04) | (0.12) | (0.06) | (0.07) |
| Age in years | 0.01*** | 0.00 | 0.01+ | 0.01+ |
|  | (0.00) | (0.02) | (0.00) | (0.01) |
| Education: Technical college entrance qualification or higher (Ref.: Lower than technical college entrance qualification) | 0.10* | 0.32* | 0.06 | 0.09 |
|  | (0.04) | (0.14) | (0.06) | (0.07) |
| Household net income: above median (Ref.: below median) | 0.16*** | 0.16 | 0.21** | 0.13+ |
|  | (0.04) | (0.11) | (0.07) | (0.07) |
| Marital status: Married, living together with spouse (Ref.: Single/Widowed/Divorced/Married, not living together with spouse) | -0.13** | -0.07 | -0.12+ | -0.12 |
|  | (0.04) | (0.12) | (0.06) | (0.08) |
| Meat consumption: - About 1 time a month (Ref.: Never) | -0.30+ | -0.68* | -0.50* | 0.24 |
|  | (0.16) | (0.32) | (0.25) | (0.30) |
| - 2-3 times a month | -0.42*** | -0.55* | -0.48* | -0.36 |
|  | (0.12) | (0.24) | (0.19) | (0.25) |
| - 1-2 times per week | -0.34** | -0.29 | -0.47** | -0.29 |
|  | (0.11) | (0.21) | (0.17) | (0.24) |
| - 3-4 times per week | -0.35** | -0.18 | -0.47** | -0.33 |
|  | (0.11) | (0.21) | (0.17) | (0.24) |
| - Every day or almost every day | -0.46*** | -0.29 | -0.58** | -0.49+ |
|  | (0.12) | (0.23) | (0.18) | (0.26) |
| Alcohol consumption: - About 1 time a month (Ref.: Never) | 0.21** | 0.06 | 0.31** | 0.13 |
|  | (0.07) | (0.19) | (0.11) | (0.12) |
| - 2-3 times a month | 0.19** | -0.01 | 0.23* | 0.23+ |
|  | (0.07) | (0.16) | (0.09) | (0.12) |
| - 1-2 times per week | 0.27*** | 0.16 | 0.32*** | 0.27* |
|  | (0.06) | (0.15) | (0.09) | (0.11) |
| - 3-4 times per week | 0.22*** | -0.05 | 0.26** | 0.27* |
|  | (0.07) | (0.19) | (0.09) | (0.11) |
| - Every day or almost every day | 0.23** | 0.30 | 0.23* | 0.31* |
|  | (0.08) | (0.33) | (0.12) | (0.13) |
| Frequency of sports activities: - About 1 time a month (Ref.: Never) | 0.02 | -0.49** | 0.18* | 0.00 |
|  | (0.07) | (0.18) | (0.09) | (0.13) |
| - 2-3 times a month | 0.18** | -0.07 | 0.41*** | -0.07 |
|  | (0.06) | (0.17) | (0.09) | (0.12) |
| - 1-2 times per week | 0.32*** | 0.12 | 0.44*** | 0.20* |
|  | (0.05) | (0.15) | (0.08) | (0.09) |
| - 3-4 times per week | 0.49*** | 0.56** | 0.66*** | 0.24* |
|  | (0.07) | (0.19) | (0.10) | (0.11) |
| - Every day or almost every day | 0.62*** | 0.50* | 0.86*** | 0.38** |
|  | (0.08) | (0.23) | (0.13) | (0.12) |
| Satisfaction with health (from 1 = very unsatisfied to 7 = very satisfied) | 0.41*** | 0.47*** | 0.41*** | 0.39*** |
|  | (0.02) | (0.06) | (0.02) | (0.03) |
|  |  |  |  |  |
| Observations | 3,222 | 459 | 1,651 | 1,112 |
| Pseudo R² | 0.12 | 0.16 | 0.13 | 0.10 |

Coefficients are displayed; robust standard errors in parentheses; *** p<0.001, ** p<0.01, * p<0.05, + p<0.10

Supplementary Table 2. Correlates of health comparisons. Results of multiple linear regressions (with FIML to address missings)

| Independent variables | Health comparisons – Total sample | Health comparisons – up to 39 years | Health comparisons – 40 to 64 years | Health comparisons – 65 years and over |
| --- | --- | --- | --- | --- |
|  |  |  |  |  |
| Sex: Female (Ref.: Male) | -0.00 | -0.17* | -0.03 | 0.10+ |
|  | (0.03) | (0.07) | (0.04) | (0.06) |
| Age in years | 0.01*** | 0.00 | 0.01* | 0.01* |
|  | (0.00) | (0.01) | (0.00) | (0.01) |
| Education: Technical college entrance qualification or higher (Ref.: Lower than technical college entrance qualification) | 0.09** | 0.12 | 0.07 | 0.09 |
|  | (0.03) | (0.08) | (0.04) | (0.06) |
| Household net income: above median (Ref.: below median) | 0.13*** | 0.13+ | 0.16** | 0.11+ |
|  | (0.03) | (0.07) | (0.05) | (0.06) |
| Marital status: Married, living together with spouse (Ref.: Single/Widowed/Divorced/Married, not living together with spouse) | -0.10** | -0.07 | -0.08+ | -0.09 |
|  | (0.03) | (0.07) | (0.04) | (0.06) |
| Meat consumption: - About 1 time a month (Ref.: Never) | -0.22* | -0.33+ | -0.37* | 0.09 |
|  | (0.11) | (0.20) | (0.17) | (0.22) |
| - 2-3 times a month | -0.28*** | -0.30* | -0.27* | -0.30+ |
|  | (0.08) | (0.15) | (0.13) | (0.18) |
| - 1-2 times per week | -0.21** | -0.15 | -0.29* | -0.19 |
|  | (0.08) | (0.13) | (0.12) | (0.17) |
| - 3-4 times per week | -0.25** | -0.10 | -0.31** | -0.23 |
|  | (0.08) | (0.13) | (0.12) | (0.18) |
| - Every day or almost every day | -0.33*** | -0.14 | -0.40** | -0.40* |
|  | (0.08) | (0.13) | (0.13) | (0.19) |
| Alcohol consumption: - About 1 time a month (Ref.: Never) | 0.15** | -0.03 | 0.24** | 0.13 |
|  | (0.05) | (0.11) | (0.08) | (0.10) |
| - 2-3 times a month | 0.15** | -0.00 | 0.18** | 0.21* |
|  | (0.05) | (0.10) | (0.07) | (0.10) |
| - 1-2 times per week | 0.20*** | 0.08 | 0.24*** | 0.24** |
|  | (0.05) | (0.10) | (0.06) | (0.09) |
| - 3-4 times per week | 0.19*** | 0.00 | 0.20** | 0.30** |
|  | (0.05) | (0.12) | (0.07) | (0.09) |
| - Every day or almost every day | 0.16** | 0.14 | 0.19* | 0.23* |
|  | (0.06) | (0.21) | (0.09) | (0.10) |
| Frequency of sports activities: - About 1 time a month (Ref.: Never) | 0.03 | -0.28** | 0.14* | 0.03 |
|  | (0.05) | (0.10) | (0.06) | (0.10) |
| - 2-3 times a month | 0.16** | 0.00 | 0.30*** | -0.01 |
|  | (0.05) | (0.10) | (0.06) | (0.10) |
| - 1-2 times per week | 0.25*** | 0.07 | 0.33*** | 0.19** |
|  | (0.04) | (0.09) | (0.05) | (0.07) |
| - 3-4 times per week | 0.38*** | 0.37** | 0.47*** | 0.24** |
|  | (0.05) | (0.12) | (0.07) | (0.09) |
| - Every day or almost every day | 0.47*** | 0.33* | 0.65*** | 0.32*** |
|  | (0.06) | (0.15) | (0.09) | (0.10) |
| Satisfaction with health (from 1 = very unsatisfied to 7 = very satisfied) | 0.32*** | 0.30*** | 0.31*** | 0.33*** |
|  | (0.01) | (0.03) | (0.02) | (0.02) |
| Constant | 0.81*** | 1.47*** | 1.02*** | 0.73 |
|  | (0.12) | (0.38) | (0.22) | (0.49) |
|  |  |  |  |  |
| Observations | 3,876 | 555 | 1,934 | 1,345 |
| R² | 0.28 | 0.32 | 0.31 | 0.24 |

Beta-coefficients (unstandardized) are displayed; robust standard errors in parentheses; *** p<0.001, ** p<0.01, * p<0.05, + p<0.10
